# Evaluation of Overwintering Performance of Threeline Grunt (*Parapristipoma trilineatum*) in Sea Cages Along the Southern Coast of Korea

**DOI:** 10.3390/ani16182841

**Published:** 2026-09-09

**Authors:** Seong Il Baek, Yong-Woon Ryu, Kang-Hee Im, Kang-Woong Kim, Jin Woo Park

**Affiliations:** Subtropical Fisheries Research Institute, National Institute of Fisheries Science, Jeju 63610, Republic of Korea; qortjddlff@naver.com (S.I.B.); ryuyw@korea.kr (Y.-W.R.); imk0408@naver.com (K.-H.I.);

**Keywords:** threeline grunt, inland tank, sea cage, overwintering, winter temperature, antioxidant response

## Abstract

To evaluate the overwintering performance of threeline grunt (*Parapristipoma trilineatum*), field experiments were conducted on 3000 subadults and 5000 juveniles reared in sea cages along the southern coast of Korea (SC group), and 600 subadults and 700 juveniles reared in inland tanks on Jeju Island (IT group), under thermally suitable overwintering conditions. Fish were sampled from January to April. The SC group maintained acceptable survival rates of 77.2% in subadults and 75.8% in juveniles. Body weight and standard length in the SC group did not significantly change during overwintering; condition factor and hepatosomatic index varied at specific sampling points. The SC group juveniles exhibited lower plasma glucose levels than the IT group ones. Overwintering in sea cages influenced body composition, characterized by reduced crude protein and increased crude lipid content. Plasma cortisol levels in the SC group juveniles increased significantly in January, whereas hepatic heat shock protein-70 levels showed no distinct changes. Principal component analysis revealed no clustering among groups. Physiological variations in the SC group reflected seasonal adaptation, evidenced by stable survival and no strong coordinated changes among physiological responses. These findings highlight the potential of threeline grunt to overwinter in sea cages along the southern coast of Korea.

## Simple Summary

Threeline grunt (*Parapristipoma trilineatum*) is a promising candidate species for diversifying aquaculture in Korea under global warming and rising water temperatures. In this study, we investigated biochemical, body composition, antioxidant, and stress-related responses of *P. trilineatum* during the winter season. Fish reared in sea cages maintained stable survival, whereas changes in other physiological parameters suggested seasonal adjustments to the overwintering environment rather than severe stress. These findings indicate that threeline grunt may overwinter in southern Korean coastal waters, supporting its potential as a candidate species for sea-cage aquaculture.

## 1. Introduction

Global warming has caused substantial alterations in marine ecosystems, with rising sea surface temperature (SST) emerging as one of its most direct and evident symptoms [1,2]. According to the Intergovernmental Panel on Climate Change (IPCC), the rate of ocean warming has more than doubled since 1993, underscoring the accelerating impact of global warming on marine environments [3]. In the Republic of Korea (hereafter referred to as Korea), from 1968 to 2024, the long-term trend for SST in coastal waters increased by approximately 1.58 °C, which is approximately 2.1-times higher than the global average (0.74 °C) [4]. Furthermore, since the 2010s, the rate of SST increase during summer has intensified markedly, which is indicative of a pronounced and accelerated upward trend [5,6]. These changes in SST around the Korean Peninsula are expected to substantially impact the aquaculture production, leading to economic losses in Korea.

The increasing frequency and prolonged duration of high SST in the coastal waters of Korea have exacerbated physiological and metabolic stress in conventionally farmed fish species, such as olive flounder (*Paralichthys olivaceus*), Korean rockfish (*Sebastes schlegelii*), and red seabream (*Pagrus major*). Chronic exposure to water temperatures above 28 °C can lead to growth retardation, reduced antioxidant and immune responses, and increased mortality in these species [7,8]. Consistent with this, Kim et al. [9] reported that the optimal temperature range for growth of these species is 18–25 °C, whereas their tolerable range is 28–32 °C. Notably, in 2024, along the southern coast of Korea, SST exceeding 28 °C persisted until late September, resulting in mass mortality across aquaculture farms. The economic losses attributed to high temperature-induced mortality were estimated at approximately USD 97 million (KRW 143 billion; 1 USD = KRW 1480). Consequently, global warming–induced increases in SST have become a critical threat to aquaculture in Korea, necessitating the development of new aquaculture species with a high tolerance to elevated water temperatures to cope with climate change and minimize economic losses.

Threeline grunt (*Parapristipoma trilineatum*), originating from southern Japan in the South China Sea, inhabits shallow coastal areas up to approximately 60 m depth [10]. Although economically important in Japan and China as both a fishery resource and aquaculture species, threeline grunt is yet to be commercially farmed in Korea. However, it has been confirmed to grow successfully at water temperatures of approximately 30 °C, which indicates that it can serve as a thermally resilient aquaculture species with higher thermal tolerance than the currently farmed species, with potential to adequately cope with rising water temperatures under climate change [11]. Although basic physiological and reproductive studies have been conducted on this species [11,12], studies and data required to evaluate the feasibility for its aquaculture in coastal environments in Korea prior to its commercial application remain limited. In addition, given that this species naturally inhabits relatively warm environments, to date, no studies have reported normal growth or successful year-round aquaculture in the comparatively lower-temperature coastal waters of mainland Korea.

Despite the warming trend, seasonal fluctuations in temperature of the waters surrounding Korea are substantial, with winter temperatures dropping to around 10 °C [6], which may impair physiological functions and increase mortality in fish species that normally inhabit comparatively warmer waters. Therefore, evaluating the overwintering performance of threeline grunt in the waters surrounding Korea is crucial for determining the feasibility for its aquaculture. The southern coast of Korea, which accounts for approximately 90% of sea-cage aquaculture in terms of the number of farms (743) and area (760,436 m^2^), is a highly representative and suitable setting for evaluating the aquaculture potential of this species [13]. Accordingly, this study was designed to investigate the physiological responses of different-sized threeline grunt during the winter season in sea cages along the southern coast of Korea and to assess their overwintering performance by comparing them with conspecifics reared in land-based tanks on Jeju Island, where water temperatures are within the habitable thermal range for this species. The rationale for evaluating different size groups (subadult and juvenile) was that thermal tolerance of fish varies with body size [14,15]. To the best of our knowledge, this is the first study to evaluate the overwintering performance of threeline grunt and to assess its aquaculture potential under sea cage conditions.

## 2. Materials and Methods

### 2.1. Fish and Experimental Conditions

**Figure 1:**
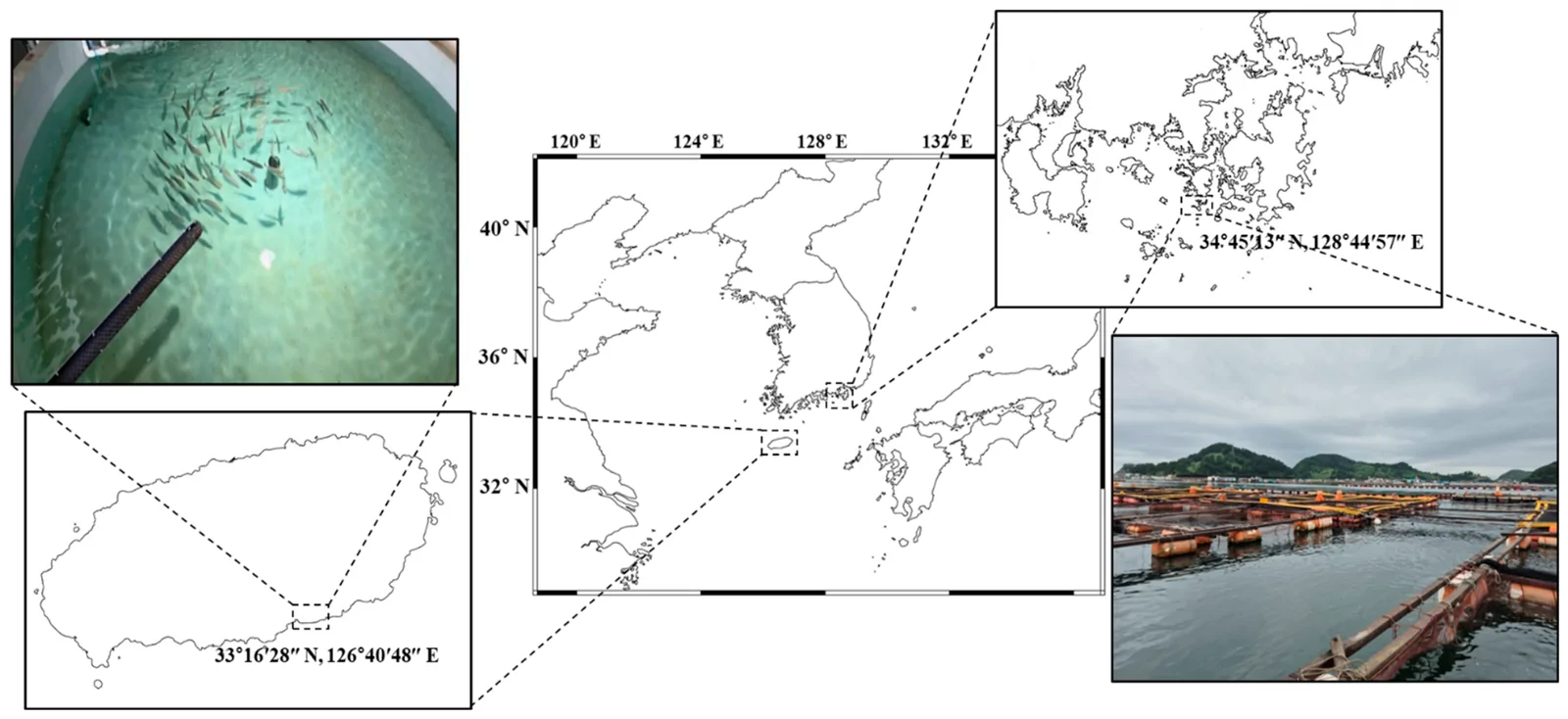
Geographical locations and overview of the inland tank (Seogwipo-si, Jeju-do, Republic of Korea) and sea cage (Tongyeong-si, Gyeongsangnam-do, Republic of Korea) experimental facilities.

A total of 3600 subadult (average weight of ca. 80.0 g) and 5700 juvenile (average weight of ca. 20.0 g) threeline grunt raised from fertilized eggs produced by captive broodstock were reared at Subtropical Fisheries Research Institute (Seogwipo-si, Jeju-do, Republic of Korea; 33°16′28″ N, 126°40′48″ E) prior to the experiment. On 19 August 2024, 3000 subadults and 5000 juveniles were transported to the southern coast of Korea (Tongyeong-si, Gyeongsangnam-do, Republic of Korea; 34°45′13″ N, 128°44′57″ E) and stocked in two 140-ton sea cages (7 m × 7 m × 5 m), corresponding to stocking densities of 1.7 and 0.7 kg/m^3^, respectively, and are hereafter referred to as the sea cage (SC) group (Figure 1). The remaining 600 subadults and 700 juveniles were stocked in two 30-ton circular tanks (7 m diameter, 0.8 m depth) at the institute at stocking densities of 1.6 and 0.5 kg/m^3^, respectively, until the end of the experiment, and are hereafter referred to as the inland tank (IT) group. For each environmental condition and size class, fish were reared in a single tank or sea cage, and no independent tank or sea cage replicates were used. Following transfer on 19 August 2024, the fish were maintained under their respective rearing conditions for approximately five months before the first experimental sampling on 22 January 2025. Fish were hand-fed a commercial extruded pellet (52% crude protein and 12% crude lipid) twice daily, until apparent satiation, throughout the rearing and experimental periods. The photoperiod and water temperature for both groups followed natural environmental conditions at each location throughout the experimental period. For the IT group, sand-filtered natural seawater was supplied at a daily exchange rate of 400%, with sufficient aeration. Water temperature was monitored daily using a YSI ProDSS (software version 1.3.35) digital water quality meter (YSI Inc., Yellow Springs, OH, USA). The tank bottoms were cleaned daily to maintain optimal water quality. For the SC group, water temperature was daily recorded using an HOBO Water Temp Pro V2 automatic water temperature measurement device (Onset Company, Bourne, MA, USA). In addition, dissolved oxygen (DO), salinity, and pH were measured using a YSI ProDSS digital water quality meter (YSI Inc., Yellow Springs, OH, USA). Total ammonia nitrogen (TAN) was determined using a spectrophotometer (DR3900, HACH Company, Loveland, CO, USA), and chlorophyll-a concentration was measured using a field fluorometer (10-AU, Turner Designs, San Jose, CA, USA). These water quality parameters were measured monthly at each sampling point during the experimental period.

### 2.2. Sampling Procedure

Sampling was conducted after a 24 h fasting period on the 20th–25th day of each month from January to April 2025. During each sampling period, 20 subadult and 40 juvenile fish were randomly selected and anesthetized with ice-chilled seawater. After anesthesia, fish body weight, total length, and standard length were measured, and the viscera and liver were weighed to calculate the weight gain, specific growth rate of weight (SGR_W_), specific growth rate of length (SGR_L_), thermal growth coefficient (TGC), daily feeding rate (DFR), condition factor (CF), viscerosomatic index (VSI), and hepatosomatic index (HSI), as follows:

Weight gain (%) = (final weight of fish − initial weight of fish)/initial weight of fish;

SGR_W_ (%/day) = (Ln final weight of fish − Ln initial weight of fish) × 100/days of feeding trial;

SGR_L_ (%/day) = (Ln final standard length of fish − Ln initial standard length of fish) × 100/days of feeding trial;

TGC = (final weight of fish^1/3^ − initial weight of fish^1/3^)/days of feeding trial/daily average water temperature × 1000;

DFR = Feed consumption (g/fish)/{[body weight (g) of previous month + body weight (g) of current month]/2 × Days} × 100;

CF (g/cm^3^) = body weight of fish (g) × 100/total length of fish (cm)^3^;

VSI (%) = viscera weight of fish (g)/body weight of fish (g) × 100;

HSI (%) = liver weight of fish (g)/body weight of fish (g) × 100.

During the final sampling in April, the remaining fish in both the inland and sea cages were counted to calculate their survival. Whole body samples from each experimental group at every sampling point were collected and stored at −80 °C for subsequent body composition analysis. In addition, liver samples were immediately collected after recording liver weight and stored at −80 °C until analysis of antioxidant stress responses. For analyzing biochemical parameters and antioxidant and stress responses in plasma and liver tissue, two subadult fish were pooled to generate one sample at each monthly sampling event, whereas five juvenile fish were pooled to generate one sample. Each pooled sample was subsequently treated as one analytical unit for the corresponding analyses.

### 2.3. Analysis of Body Composition

For body composition analysis, four whole-body pooled samples (*n* = 4) were prepared from each experimental group at each sampling point. Each pooled sample consisted of three subadult fish or five juvenile fish, with no individuals shared among pooled samples. The samples were finely homogenized and analyzed according to standard methods outlined by the Association of Official Analytical Chemists (AOAC) [16]. Moisture content was measured via oven drying at 105 °C. Crude protein content was determined using the semi-automated Kjeldahl method (Gerhardt VAP 50 OT/TT125, C. Gerhardt GmbH & Co. KG, Königswinter, Germany), with a nitrogen-to-protein conversion factor of 6.25 applied following acid digestion. Crude lipid content was analyzed using Soxhlet extraction with ethyl ether (Soxtec 2043; Foss, Hillerød, Denmark). Ash content was determined by incineration at 600 °C for 6 h in a muffle furnace (FHPX-14, Daihan Scientific Co., Ltd., Daegu, Republic of Korea).

### 2.4. Analysis of Biochemical Parameters

Blood samples were collected from the caudal vein using heparinized syringes after measuring fish body weight, total length, and standard length. The samples were centrifuged at 5000× *g* for 20 min at 4 °C to separate the plasma. The obtained plasma samples were stored at −80 °C until analysis of biochemical parameters and cortisol concentrations. Plasma aspartate aminotransferase (AST; Catalog No. 15809542), alanine aminotransferase (ALT; Catalog No. 15809554), alkaline phosphatase (ALP; Catalog No. 15809401), total cholesterol (T-CHO; Catalog No. 15809669), triglyceride (TG; Catalog No. 15809671), and glucose (GLU; Catalog No. 15809528) levels were analyzed using commercially available dry chemistry kits (Fujifilm Corp., Tokyo, Japan) with an automatic chemistry system (FUJI DRI-CHEM IMMUNO 7000i; Fujifilm Corp.).

### 2.5. Analysis of Antioxidant and Stress Responses

To assess the antioxidant and stress responses of fish, hepatic glutathione peroxidase (GPX; MBS1603287), catalase (CAT; MBS8807784), superoxide dismutase (SOD; MBS705758), malondialdehyde (MDA; MBS1601664), cortisol (MBS704055), and heat shock protein-70 (HSP-70; MBS706016) levels were measured using enzyme-linked immunosorbent assay (ELISA) kits (MyBioSource Inc, San Diego, CA, USA) with a microplate reader (Tecan, Männedorf, Switzerland). All assays were performed according to the manufacturer’s protocols.

### 2.6. Statistical Analysis

Individual fish and pooled samples were used as the statistical units, depending on the parameter analyzed, and the results are presented as means ± standard error (SE). All statistical analyses were performed using IBM SPSS Statistics version 19 (SPSS Inc., Chicago, IL, USA) for Windows. Prior to analysis, the assumptions of normality and homogeneity of variance were evaluated using the Shapiro–Wilk and Levene’s tests, respectively. Two types of statistical analyses were performed. First, a one-way analysis of variance (ANOVA) was used to evaluate the differences among months within each group. When the variances of the data were homogeneous, Tukey’s honestly significant difference (HSD) test was used as a post hoc test. When variances were not homogeneous, the Games–Howell post hoc test was used. Second, an independent *t*-test was conducted to compare the IT and SC groups at each sampling point, with significance indicated as “ns” (not significant, *p* > 0.05) and * (significant, *p* < 0.05). Principal component analysis (PCA) was performed to identify the major contributing factors and underlying patterns of the biochemical, antioxidant, and stress responses of fish. The standardized scores of the first two components, which explained the highest proportion of total variance, were used to construct the biplots. In addition, hierarchical cluster analysis using average linkage and Pearson’s correlation coefficient was performed after log_2_ transformation of the data to examine the relationships between biochemical, antioxidant, and stress responses and experimental groups. Hierarchical clustering was visualized as heatmaps using standardized (Z-score) values to facilitate the visualization of patterns and covariation among parameters across experimental groups.

## 3. Results

### 3.1. Changes in Water Temperature During the Experimental Period

**Figure 2:**
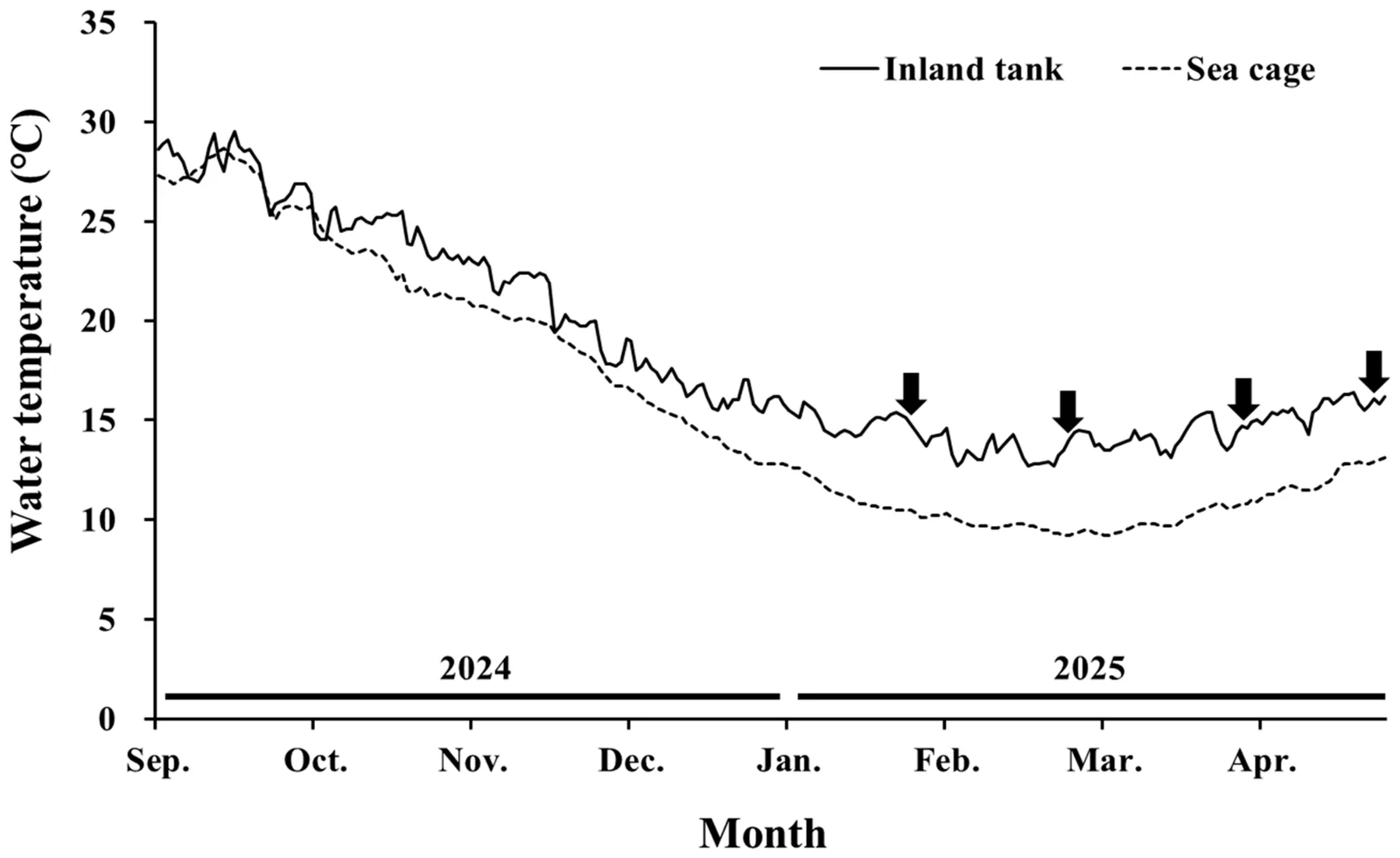
Changes in the water temperature of the inland tank and sea cage during the experimental period. The black arrows indicate the sampling dates for the threeline grunt (*Parapristipoma trilineatum*).

Changes in water temperature in the inland tank and sea cage during the experimental period are shown in Figure 2. Water temperatures in both systems decreased from September, and gradually increased from March onward. The highest and lowest water temperatures recorded in the inland tank were 29.5 °C on 16 September and 12.7 °C on 19 February, respectively, whereas those in the sea cage were 28.7 °C on 14 September and 9.2 °C on 26 February, respectively. Water temperatures in the inland tank were generally higher than those in the sea cage from November onward. The monthly average water temperatures in the inland tank and the sea cage were 27.7 and 27.1 °C in September, 24.5 and 22.8 °C in October, 21.0 and 19.4 °C in November, 16.8 and 14.6 °C in December, 13.4 and 11.3 °C in January, 13.4 and 9.7 °C in February, 14.1 and 9.9 °C in March, and 15.5 and 11.9 °C in April, respectively. DO, salinity, pH, TAN, and chlorophyll-a values ranged from 7.69 to 8.14 mg/L, from 32.05 to 33.77 psu, from 7.98 to 8.10, from 17 to 124 μg/L, and from 0.7 to 1.5 μg/L, respectively, in the sea cage, and from 6.71 to 9.37 mg/L, from 31.36 to 32.10 psu, from 7.75 to 8.07, from 88 to 196 μg/L, and from 0.2 to 0.5 μg/L, respectively, in the inland tank.

### 3.2. Survival, Growth Performance, and Feed Intake of Threeline Grunt

**Figure 3:**
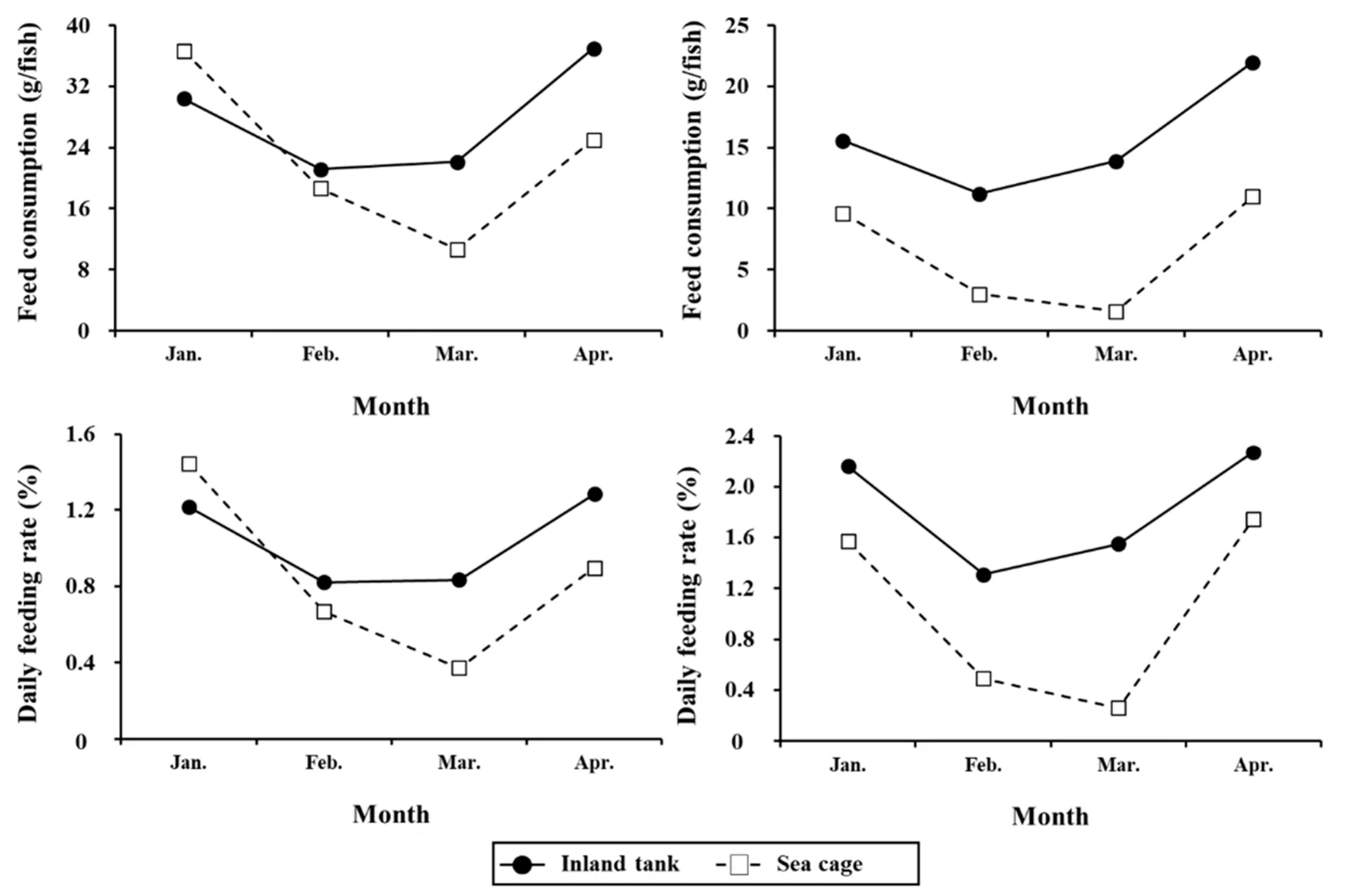
Monthly changes in feed consumption (g/fish) and daily feeding rate (%) of subadult (**left**) and juvenile (**right**) threeline grunts (*Parapristipoma trilineatum*) reared in inland and sea cages for the experimental period.

The survival rates and growth performances of the fish are presented in Table 1. In subadult fish, the IT group exhibited a survival rate of 92.0% compared with 77.2% in the SC group. In juvenile fish, the IT group showed a survival rate of 96.3%, whereas the SC group exhibited 75.8%. In both the subadult and juvenile fish, the IT group exhibited greater weight gain, SGR_W_, SGR_L_, and TGC than the SC group. For subadult fish, feed consumption and DFR in the SC group were lower than those in the IT group from February onward and remained at low levels throughout the experimental period (Figure 3). For juvenile fish, the SC group consistently maintained lower levels compared with the IT group throughout the experimental period. The SC groups exhibited the lowest feed consumption and DFR in March.

### 3.3. Biological Indices of Threeline Grunt

**Figure 4:**
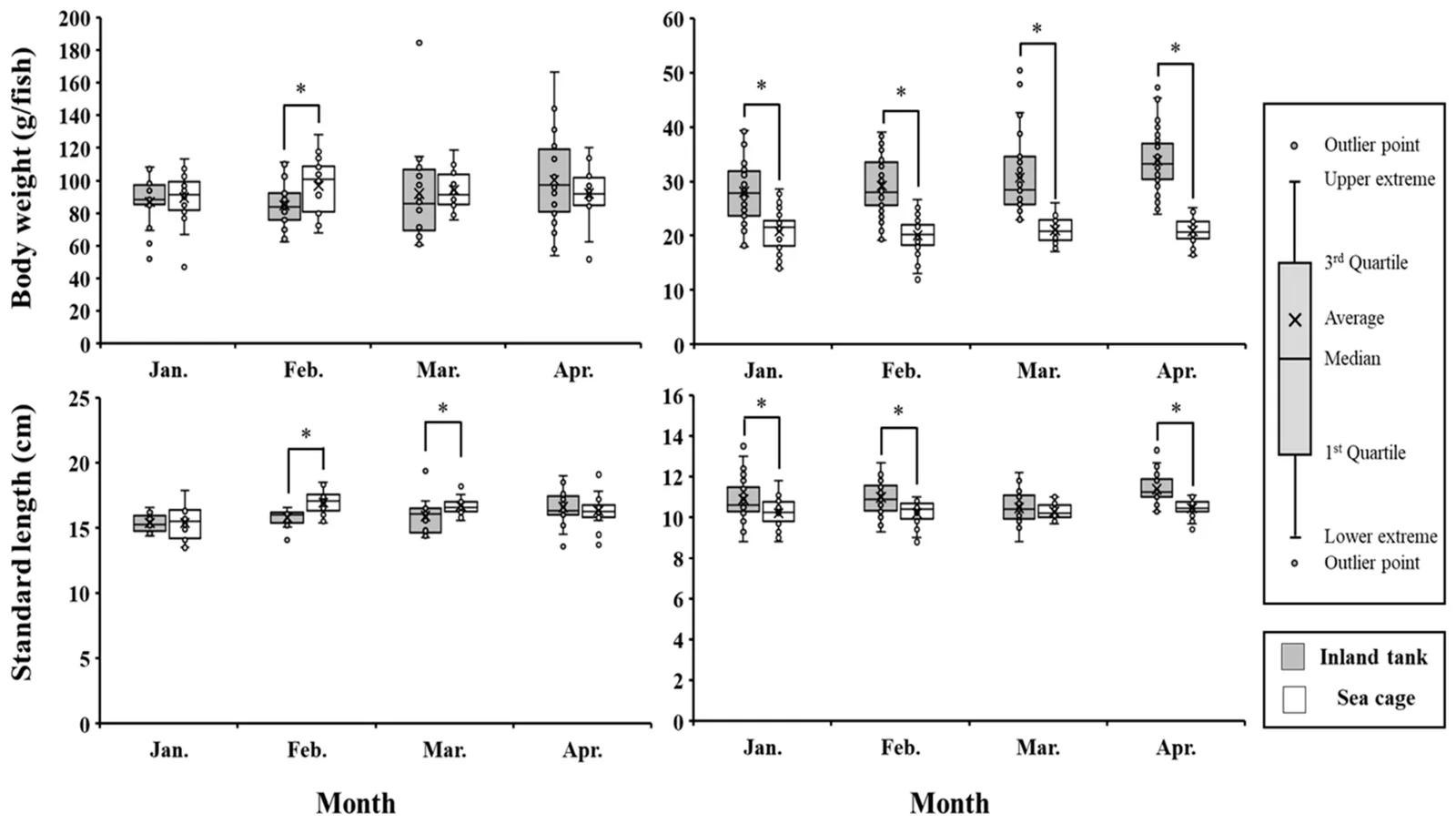
Monthly changes in body weight (g/fish) and standard length (cm) of subadult (**left**) and juvenile (**right**) threeline grunts (*Parapristipoma trilineatum*) reared in inland and sea cages during the experimental period (box–whisker diagram). The data are presented as mean ± SE (*n* = 20 for Subadult and *n* = 40 for juvenile, respectively). Asterisks (*) indicate significant (*p* < 0.05) differences between the inland tank and sea cage groups at each sampling time point.

**Figure 5:**
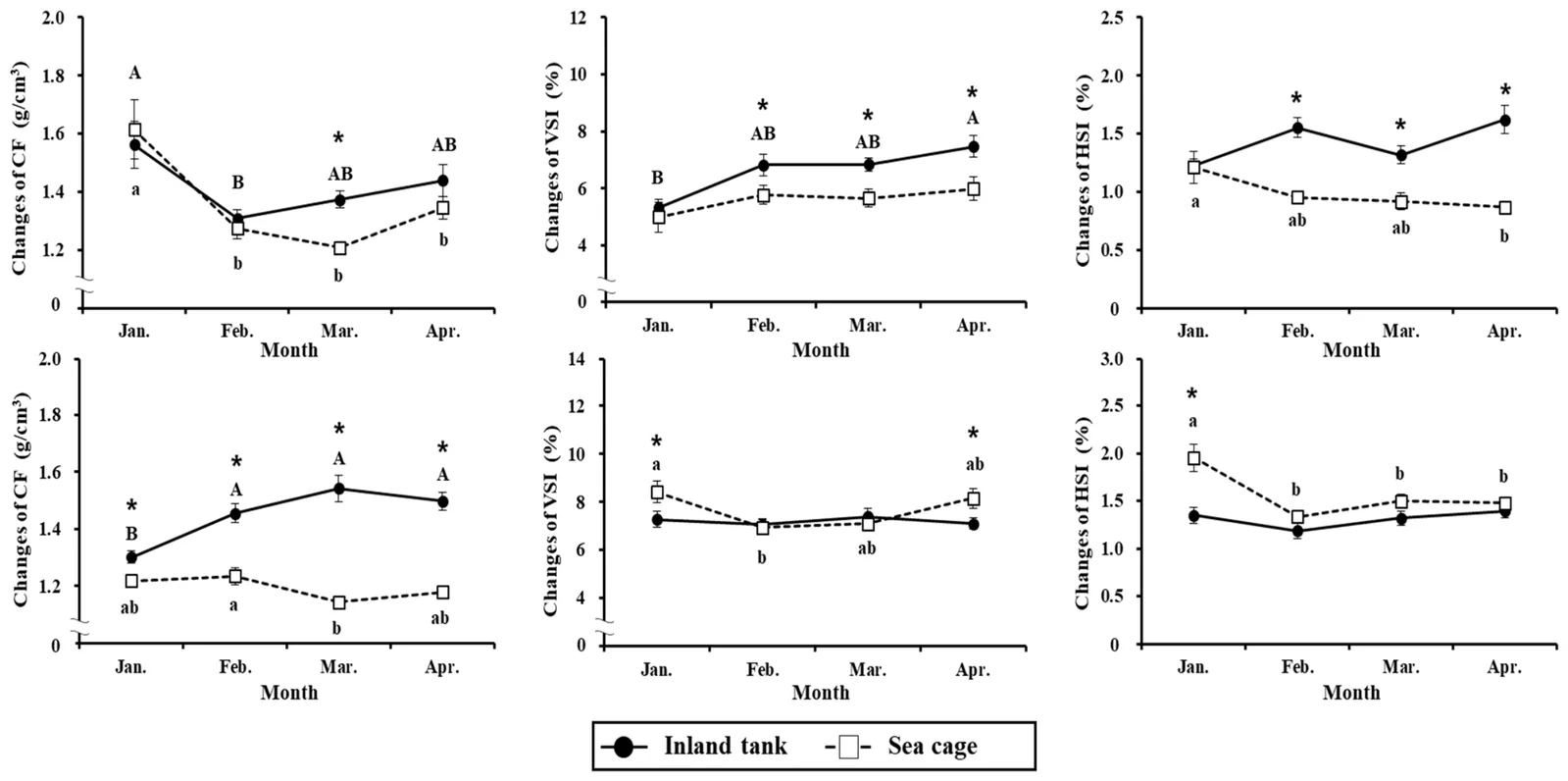
Monthly changes in biological indices of different-sized threeline grunts (*Parapristipoma trilineatum*) reared in inland and sea cages for the experimental period (upper data for subadults and lower data for juveniles). The data are presented as mean ± SE (*n* = 20). Asterisks (*) indicate significant differences (*p* < 0.05) between the inland tank and sea cage groups at each sampling time point. Values sharing the same letters (uppercase for the inland tank and lowercase for the sea cage groups) in each group were not significantly different (*p* < 0.05).

Monthly changes in body weight and standard length are shown in Figure 4. For subadult fish, the IT group exhibited significantly higher (*p* < 0.05) body weight than the SC group in February and significantly higher (*p* < 0.05) standard length in February and March. For juvenile fish, with the exception of the standard length in March, both the body weight and standard length of the IT group were significantly higher (*p* < 0.05) than those of the SC group at all sampling points. The monthly changes in CF, VSI, and HSI are shown in Figure 5. For the subadult fish, the CF of the IT group decreased until February and subsequently increased, whereas that of the SC group decreased until March and increased in April. In March, the CF of the IT group was significantly higher (*p* < 0.05) than that of the SC group. The VSI of the IT group increased throughout the experimental period, and was significantly higher (*p* < 0.05) than that of the SC group at all sampling points, except January. The HSI of the SC group decreased throughout the experimental period and was significantly lower (*p* < 0.05) than that of the IT group at all sampling points except in January. For juvenile fish, the CF of the IT group increased in February and remained significantly higher (*p* < 0.05) from February to April than that in January. The CF of the IT group was significantly higher (*p* < 0.05) than that of the SC group throughout the experimental period. No significant changes (*p* > 0.05) were observed in the VSI and HSI of the IT group during the experimental period, whereas the SC group exhibited significantly higher (*p* < 0.05) VSI and HSI in January than in February and the subsequent months. Furthermore, the SC group showed significantly higher (*p* < 0.05) VSI than the IT group in January and April and significantly higher (*p* < 0.05) HSI in April.

### 3.4. Biochemical Parameters of Threeline Grunt

**Figure 6:**
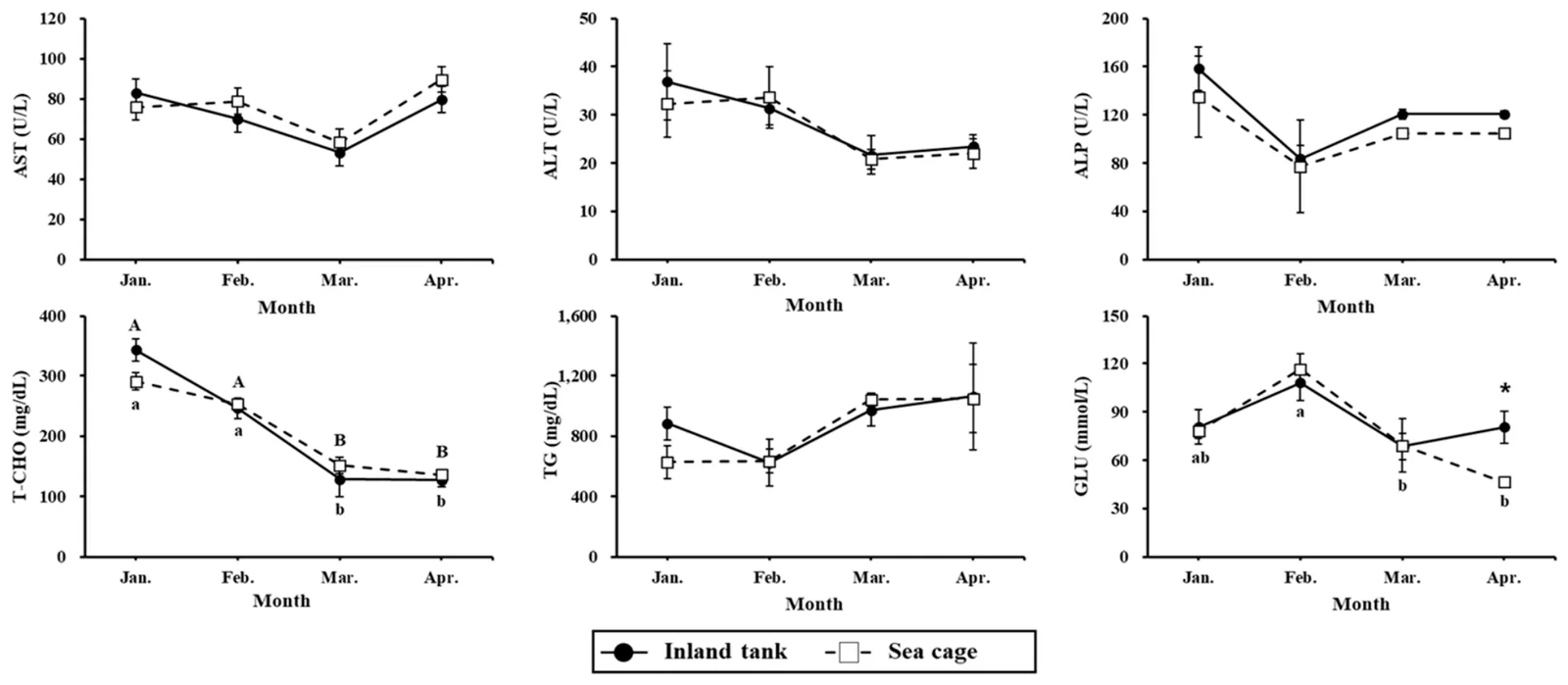
Monthly changes in biochemical parameters of a subadult threeline grunt (*Parapristipoma trilineatum*) reared inland and in sea cages during the experimental period. The data are presented as mean ± SE (*n* = 4). Asterisks (*) indicate significant differences (*p* < 0.05) between the inland tank and sea cage groups at each sampling time point. Values sharing the same letters (uppercase for the inland tank and lowercase for the sea cage groups) in each group were not significantly different (*p* < 0.05).

**Figure 7:**
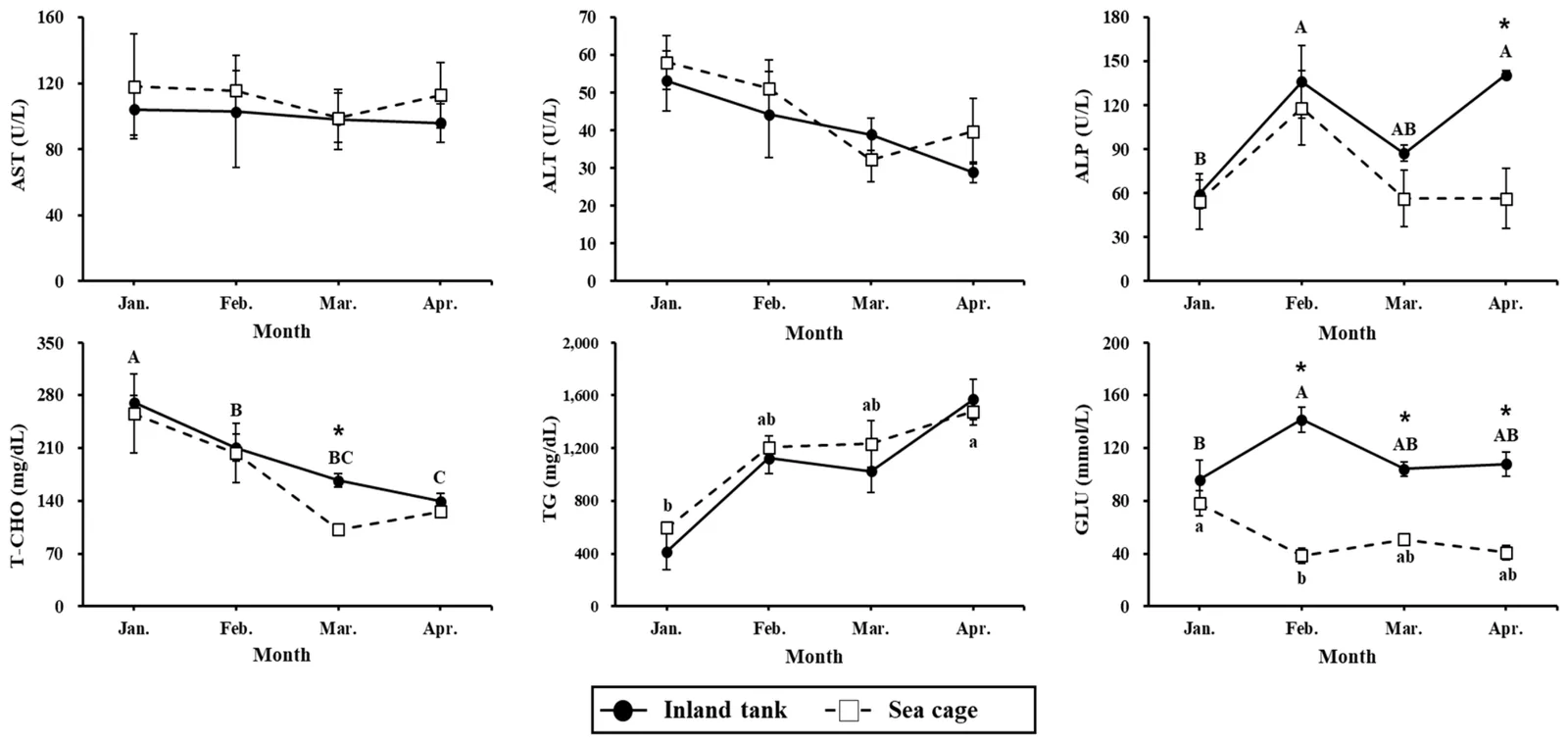
Monthly changes in biochemical parameters of a juvenile threeline grunt (*Parapristipoma trilineatum*) reared inland and in sea cages during the experimental period. The data are presented as mean ± SE (*n* = 4). Asterisks (*) indicate significant differences (*p* < 0.05) between the inland tank and sea cage groups at each sampling time point. Values sharing the same letters (uppercase for the inland tank and lowercase for the sea cage groups) in each group were not significantly different (*p* < 0.05).

The biochemical parameters of threeline grunt for each month are shown in Figure 6 and Figure 7. For subadult fish, no significant differences (*p* > 0.05) were observed in the plasma AST, ALT, ALP, and TG levels between the experimental groups. Plasma T-CHO levels in both the IT and SC groups showed decreasing trends, from 342.6 to 127.2 mg/dL and from 290.3 to 136.3 mg/dL, respectively, as the experimental period increased and were significantly lower (*p* < 0.05) in March and April than in January and February. Plasma GLU levels in the SC group were significantly higher (*p* < 0.05) in February than in March or April. The plasma GLU levels in the IT group were significantly higher (*p* < 0.05) than those in the SC group in April.

For juvenile fish, no significant differences (*p* > 0.05) were observed in plasma AST and ALT levels within or between groups during the experimental period. Plasma ALP levels in the IT group were significantly higher (*p* < 0.05) in February and April than in January, and were significantly higher (*p* < 0.05) than those in the SC group in April. Plasma T-CHO levels in the IT group showed a decreasing trend, from 270.3 to 139.2 mg/dL during the experimental period and were significantly higher (*p* < 0.05) than those in the SC group in March. Plasma TG levels in the SC group showed an increasing trend, from 596.0 to 1477.8 mg/dL, although no significant differences (*p* > 0.05) were found between the IT group during the experimental period. Compared to January, the IT group showed a significantly higher (*p* < 0.05) plasma GLU level in February, whereas that of the SC group was significantly lower (*p* < 0.05) in February. From February onward, plasma GLU levels in the IT group were significantly higher (*p* < 0.05) than those in the SC group.

### 3.5. Proximate Composition of Threeline Grunt

The proximate composition of the whole body of threeline grunt is listed in Table 2. In subadult fish, the moisture content in the SC group in February was significantly lower (*p* < 0.05) than in January and April. Moreover, the moisture content in the SC group was significantly lower (*p* < 0.05) than that in the IT group during February and March. The crude protein content in the SC group decreased until March and was significantly lower (*p* < 0.05) than that in the IT group throughout the experimental period, except in April. The crude lipid content in the SC group in February was significantly higher (*p* < 0.05) than that in January and April. Furthermore, the crude lipid content in the SC group was significantly higher (*p* < 0.05) than that in the IT group in February and March.

For juvenile fish, the moisture content in the SC group in January was significantly higher (*p* < 0.05) than that in the IT group. The crude protein content in both the IT and SC groups tended to decrease during the experimental period, and that in the SC group was significantly lower (*p* < 0.05) than that in the IT group in April. The crude lipid content in the SC group tended to increase from 11.5% to 13.8% during the experimental period; however, no significant differences (*p* > 0.05) were observed with the IT group. For both subadult and juvenile fish, no significant differences (*p* > 0.05) in the ash content were observed within or between the groups throughout the experimental period.

### 3.6. Antioxidant and Stress Responses of Threeline Grunt

**Figure 8:**
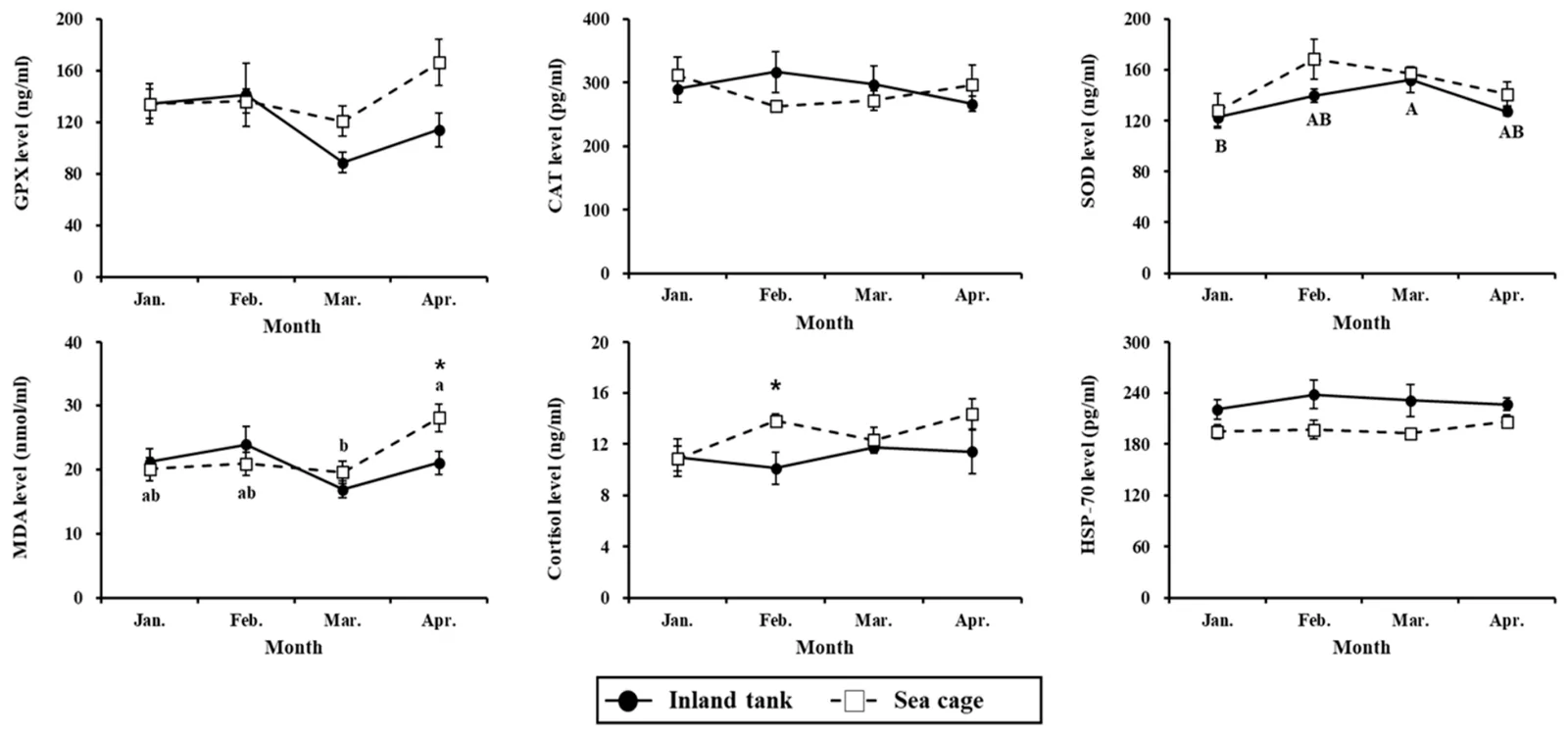
Changes in antioxidant and stress responses of a subadult threeline grunt (*Parapristipoma trilineatum*) reared inland and in sea cages during the experimental period. The data are presented as mean ± SE (*n* = 4). Asterisks (*) indicate significant differences (*p* < 0.05) between the inland tank and sea cage groups at each sampling time point. Values sharing the same letters (uppercase for the inland tank and lowercase for the sea cage groups) in each group were not significantly different (*p* < 0.05).

**Figure 9:**
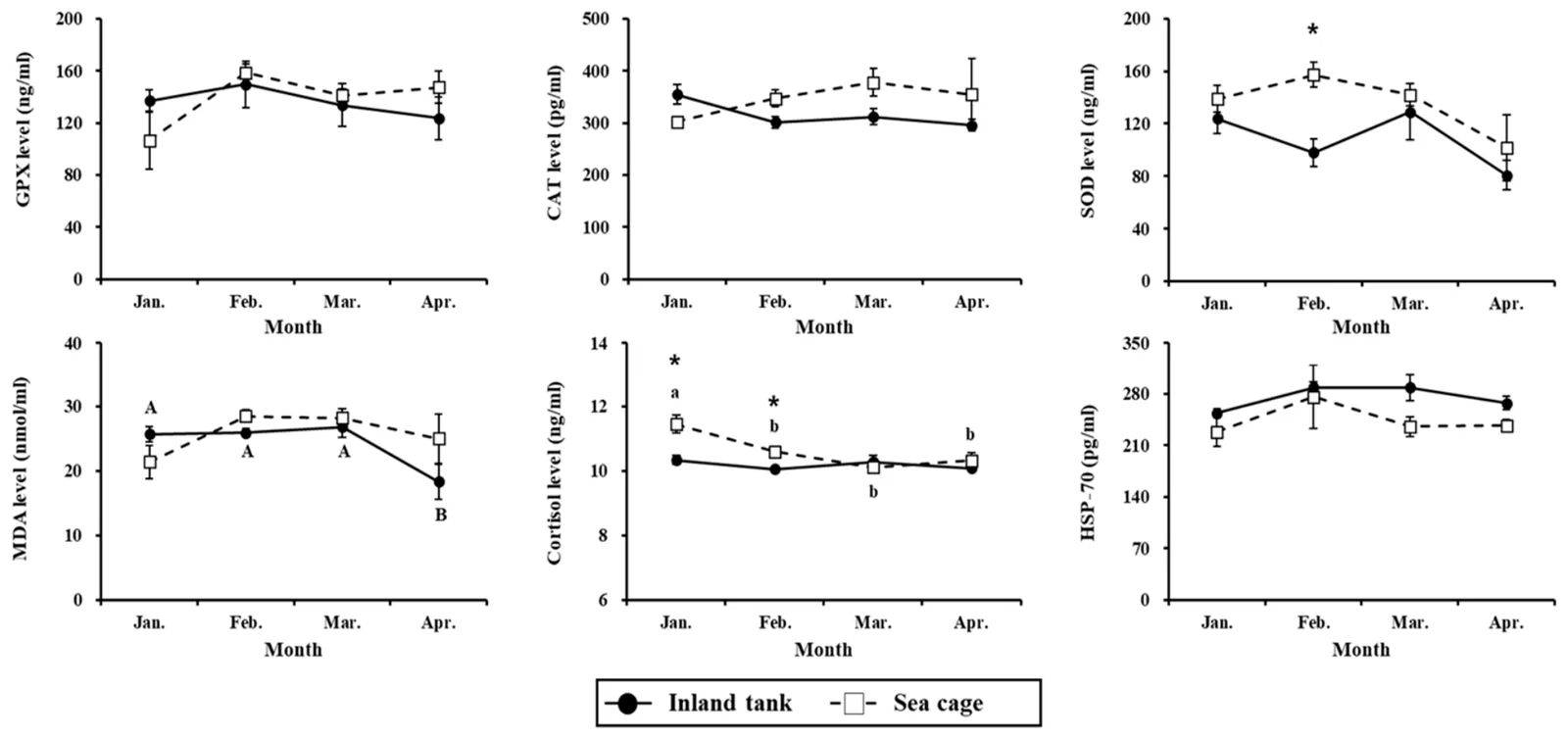
Changes in antioxidant and stress responses of juvenile threeline grunt (*Parapristipoma trilineatum*) reared in inland and sea cages during the experimental period. The data are presented as mean ± SE (*n* = 4). Asterisks (*) indicate significant differences (*p* < 0.05) between the inland tank and sea cage groups at each sampling time point. Values sharing the same letters (uppercase for the inland tank and lowercase for the sea cage groups) in each group were not significantly different (*p* < 0.05).

The antioxidant and stress responses of threeline grunt are shown in Figure 8 and Figure 9, respectively. For subadult fish, no significant differences (*p* > 0.05) were observed in hepatic GPX, CAT, or HSP-70 levels between the experimental groups throughout the experimental period. In the IT group, hepatic SOD levels in March were significantly higher (*p* < 0.05) than those in January, although no significant differences (*p* > 0.05) were detected between the IT and SC groups during the experimental period. In April, the SC group exhibited a significant increase (*p* < 0.05) in hepatic MDA levels compared to that in March, and it was also significantly higher (*p* < 0.05) than that in the IT group. In February, the plasma cortisol level in the SC group was significantly higher (*p* < 0.05) than that in the IT group.

For juvenile fish, no significant differences (*p* > 0.05) in hepatic GPX, CAT, or HSP-70 levels between the experimental groups were observed during the experimental period. Hepatic SOD levels in the SC group in February were significantly higher (*p* < 0.05) than those in the IT group were. In April, the IT group exhibited a significant increase (*p* < 0.05) in hepatic MDA levels compared with the other months, although no significant difference (*p* > 0.05) was found between the IT and SC groups. The plasma cortisol levels in the SC group tended to decrease until March, but remained significantly higher (*p* < 0.05) than those in the IT group in January and February.

### 3.7. PCA of Biochemical, Antioxidant, and Stress-Related Parameters of Threeline Grunt

**Figure 10:**
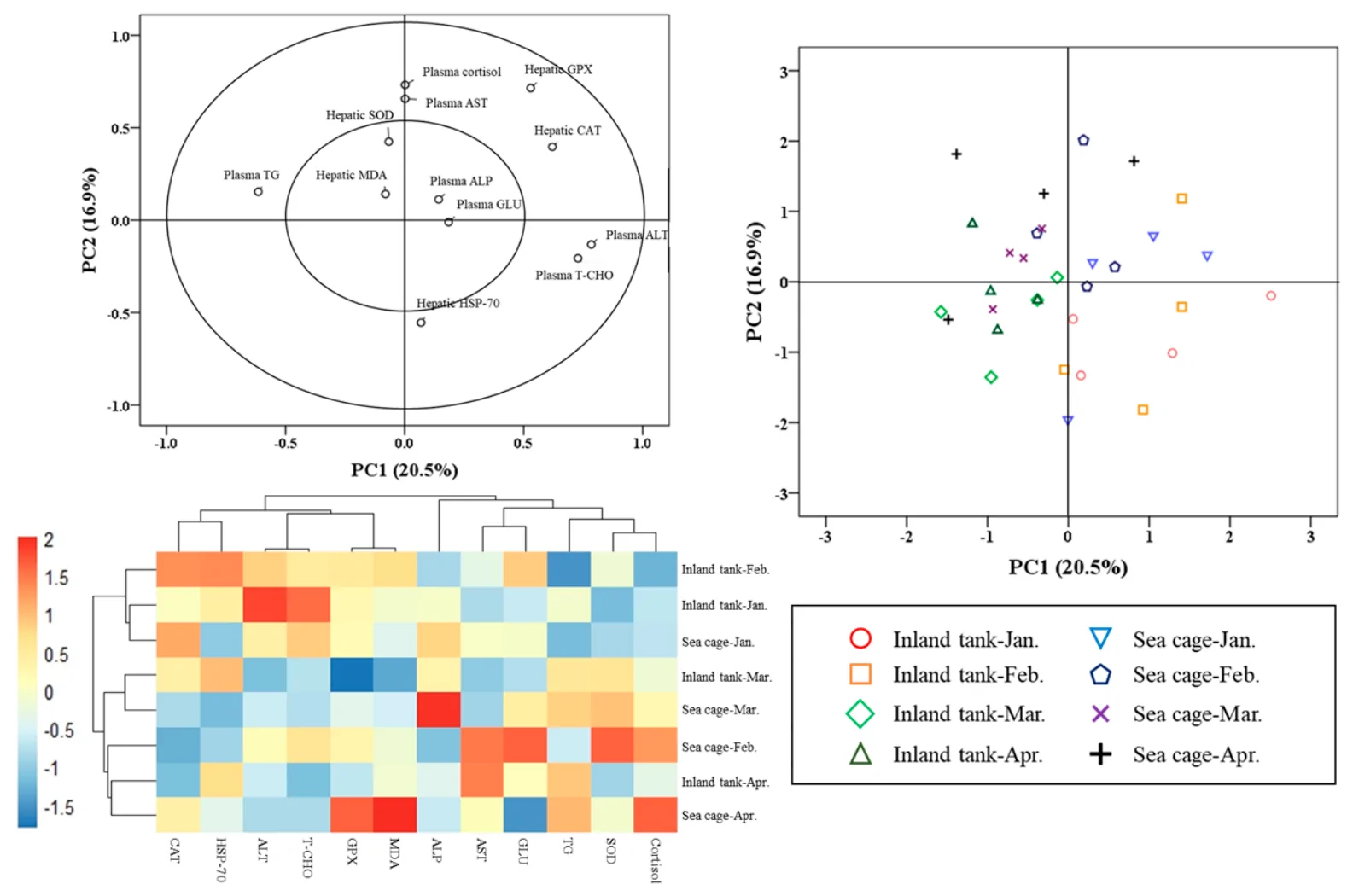
Principal component analysis (PCA) score plot (**top left**), hierarchical clustering heatmap (**bottom left**), and PCA loading plot (**right**) of biochemical, antioxidant, and stress-related parameters in subadult threeline grunt.

**Figure 11:**
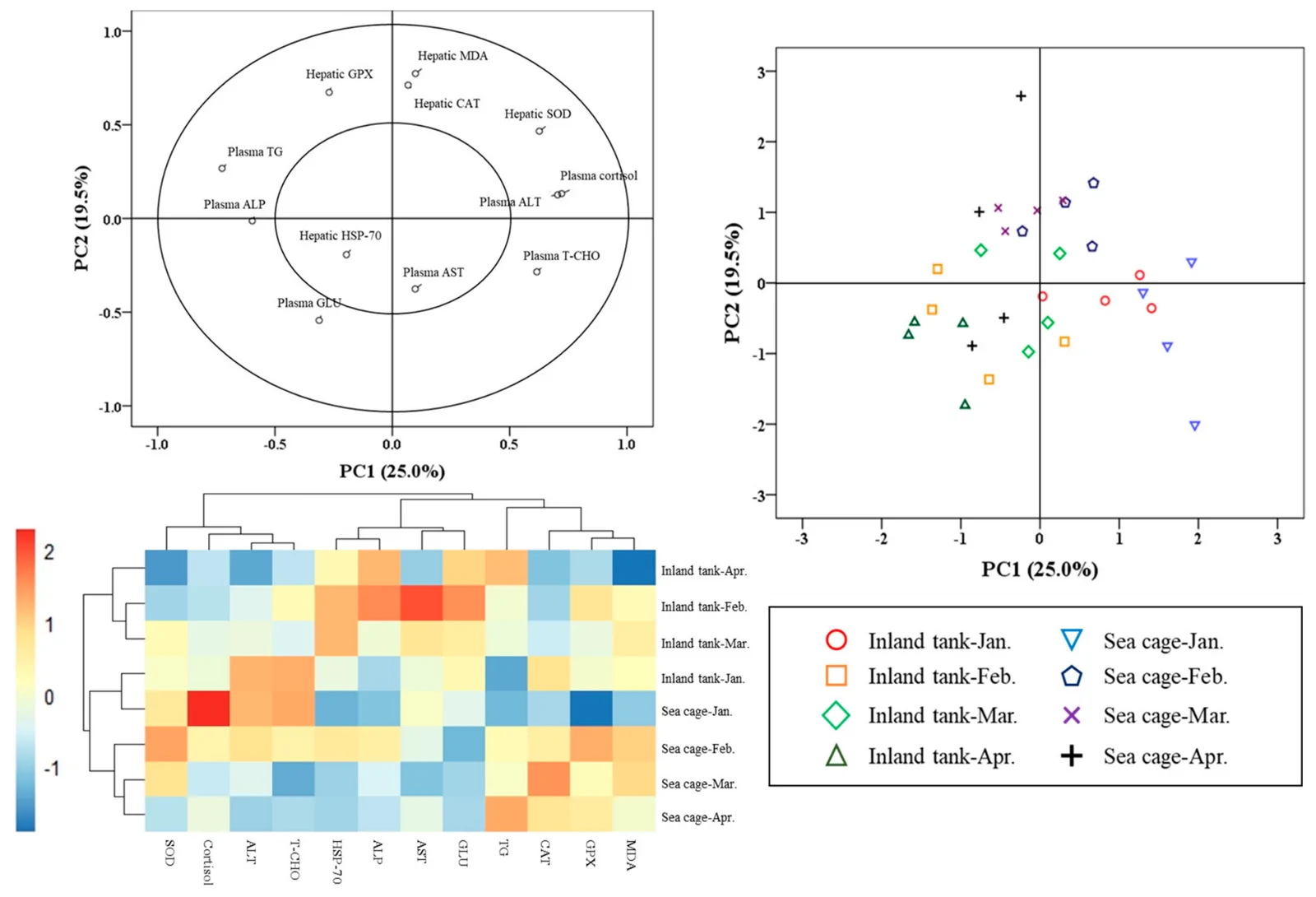
Principal component analysis (PCA) score plot (**top left**), hierarchical clustering heatmap (**bottom left**), and PCA loading plot (**right**) of biochemical, antioxidant, and stress-related parameters in juvenile threeline grunt.

The PCA scores for threeline grunt are presented in Figure 10 and Figure 11. For subadult fish, 37.4% of the variation in the PCA model was explained by the first principal (PC1, 20.5%) and second principal (PC2, 16.9%) components. The significant variables (absolute loading value > 0.5) in PC1 were plasma ALT, T-CHO, and TG as well as hepatic GPX and CAT, whereas plasma cortisol, AST, and hepatic HSP70 were the main contributors to PC2. In the score plot, no clear clustering of the antioxidant and stress responses was observed among the experimental groups. In hierarchical clustering heatmap, only three experimental groups (the IT group in January and February and the SC group in January) clustered closely, whereas the remaining groups were separately distributed, with no clear clustering pattern among physiological parameters or rearing environments.

For juvenile fish, 44.5% of the variation in the PCA model was explained by PC1 (25.0%) and PC2 (19.5%). The major variables contributing to PC1 were plasma ALP, ALT, T-CHO, TG, cortisol, and hepatic SOD, whereas plasma GLU, hepatic CAT, GPX, and MDA were identified as major contributors to PC2. Similarly, no clear clustering of the antioxidant and stress responses among the experimental groups was observed in the score plot. In the hierarchical clustering heatmap, the IT and SC groups formed separate clusters from February to April, whereas the physiological parameters did not form a consistent cluster.

## 4. Discussion

The present study is the first to comparatively evaluate the physiological aspects of the overwintering performance for threeline grunt in sea cages in the coastal waters of Korea and inland tanks on Jeju Island. Analyzing long-term physiological changes in fish in aquaculture facilities provides more information than assessing short-term responses under laboratory conditions, as it provides a comprehensive understanding of cumulative responses, survival, and adaptability to environmental conditions [17,18]. Although differences in other water quality parameters (DO, salinity, pH, TAN, and chlorophyll-a) cannot be entirely excluded, all the parameters were within acceptable ranges for fish culture [19,20], suggesting that water temperature was likely the primary environmental factor affecting physiological responses. Fish reared at relatively high water temperatures, within an acceptable thermal range, generally exhibit enhanced growth performance and survival. However, the magnitude of these responses varies with body size, even under similar environmental conditions [21,22,23]. During the experimental period, the IT group, which was reared at relatively higher water temperatures than the SC group, exhibited a higher survival rate, weight gain, SGR_W_, and SGR_L_. The reduced growth observed in the SC group was likely attributable to both increased energy expenditure for maintenance under winter condition and reduced feed intake associated with temperature-induced suppression of digestive and metabolic processes, with these effects potentially being more pronounced for smaller fish and thereby contributing to the greater growth suppression observed for juveniles [24]. Survival rate is an important parameter for evaluating overwintering performance, with rates above 70% generally considered acceptable [21,25]; given that major fish species in grow-out stage cultured in the region typically show winter survival rates of 70–75%, the survival rates of threeline grunt (75.8–77.2%) can also be considered acceptable. The SC group exhibited relatively low survival rates compared with the IT group. However, from the perspective of overwintering viability, the survival rate in the sea cage remained within an acceptable range, highlighting the potential of this species as a promising candidate for aquaculture in this region. The lower TGC observed for the SC group than for the IT group was likely attributable to increased metabolic demands for basal maintenance and voluntary fasting under prolonged low-temperature conditions, resulting in reduced growth efficiency. Notably, the pronounced difference in TGC (approximately 40-fold) observed for juvenile fish suggests that smaller fish experience a disproportionately greater metabolic burden relative to growth, even under similar environmental conditions. Past studies also demonstrated that seasonal variation as well as artificially reduced water temperature can reduce TGC in fish, likely through decreased feed intake and suppressed metabolic activity [26,27,28].

Biological indices (CF, VSI, and HSI) are key indicators of the physiological and nutritional status of fish under changing environmental conditions [29,30]. In this study, marked differences in these indices between the SC and IT groups, particularly the significant reduction in cold-exposed juveniles compared with subadults, highlight size-dependent variations in thermal sensitivity. Although temperature-induced responses in biological indices can vary depending on species and experimental conditions [31,32,33,34], our findings clearly demonstrate that smaller juveniles experience greater physiological strain under prolonged winter temperature conditions than larger subadults.

Biochemical parameters are widely regarded as important indicators of fish health under various environmental conditions. AST and ALT are widely distributed enzymes primarily localized in the mitochondria of fish cells, and their plasma activities may increase in response to tissue damage or the disruption of cellular function [35,36]. Accordingly, chronic exposure to low water temperatures reportedly elevates plasma AST and ALT activities in discus fish (*Symphysodon aequifasciatus*), Nile tilapia (*Oreochromis niloticus*), and hybrid sturgeon (*Acipenser baerii* × *A. schrenckii*), presumably due to thermal stress–induced hepatic damage [37,38,39]. However, plasma AST and ALT showed no significant difference or consistent trend between the IT and SC groups. This suggested that hepatic cellular integrity and basal liver function were not markedly impaired by winter temperature exposure during the experimental period. ALP plays an important role in membrane transport and skeletal mineralization and contributes to immune defense by facilitating pathogen recognition and phagocytosis through modifications of pathogen surface structures [40]. Although ALP activity generally increases under stressful conditions, it is reportedly suppressed when metabolic demands exceed physiological capacity [35,41] or when stress is prolonged [42]. In the present study, no significant differences in plasma ALP activity were detected in adult fish. In contrast, the juvenile fish in the SC group exhibited significantly lower ALP activity than those in the IT group in April. This reduction may partially reflect the cumulative physiological stress associated with prolonged exposure to comparatively low water temperatures under sea cage conditions, which could have suppressed metabolic or immune functions in juvenile fish.

No significant differences in T-CHO and TG levels were observed between the experimental groups at any sampling point, except for the juvenile fish in March. Nevertheless, T-CHO exhibited a decreasing trend over the experimental period, whereas TG showed an increasing trend, particularly in juvenile fish, suggesting a distinct physiological regulation of these lipid parameters under prolonged winter temperature conditions. T-CHO primarily serves as a structural component of cell membranes and lipoproteins, contributing to membrane stability, whereas TG represent the main form of lipid energy storage that can be mobilized to meet metabolic demands [43]. Under low-temperature conditions, TG synthesis and secretion increase as a compensatory response to elevated energy requirements, and glycerol released from TG hydrolysis can be converted into GLU and utilized as an energy source [44]. Similar TG responses have been reported in *Hoplosternum littorale*, pufferfish (*Takifugu fasciatus*), and Ya-fish (*Schizothorax prenanti*) exposed to low temperatures over varying durations [29,44,45,46]. In contrast, the decreasing trend in plasma T-CHO levels may be associated with reduced cholesterol synthesis during long-term cold adaptation. Robertson and Hazel [47] reported that cold acclimation in rainbow trout (*Oncorhynchus mykiss*) was accompanied by lower cholesterol-to-phospholipid ratios in tissue plasma membranes, suggesting a reduced metabolic requirement for cholesterol to maintain membrane fluidity at low temperatures. Similar species-specific responses have been reported in other fish species [44,48], but the underlying mechanisms remain unclear. Plasma GLU is a primary oxidative substrate for essential physiological activities in fish, with hepatic glycogen acting as an immediately mobilizable energy buffer during stress [29,49]. Although acute cold exposure typically triggers transient hyperglycemia via accelerated glycogenolysis, chronic low-temperature exposure in the SC group resulted in significantly suppressed plasma glucose levels, particularly in juvenile fish. This glycemic decline suggested progressive exhaustion of endogenous glycogen reserves under prolonged cold stress, which was apparently further exacerbated by severely reduced feed intake in the SC group. Such biphasic dynamics occurs in *Hoplosternum littorale*, where short-term cold exposure initially elevates blood GLU and is followed by a marked reduction upon extended exposure [29]. Although hepatic glycogen content was not directly quantified in the present study, the concurrent decrease in HSI observed in the SC group after January may reflect changes in hepatic energy storage associated with the progressive mobilization of endogenous glycogen reserves.

During periods of restricted feed intake associated with low water temperatures, fish rely on the sequential mobilization of endogenous energy reserves to sustain essential metabolic requirements. Lipids serve as a vital energy source through activated lipolysis and mitochondrial β-oxidation [50]. However, as cold exposure persists and energy limitations continue, mitochondrial substrate utilization shifts, leading to increased contribution of protein catabolism to satisfy energy demands. This protein degradation is primarily driven by activated proteolytic pathways, which mobilize body proteins to supply amino acids for gluconeogenesis and TCA cycle intermediates [51]. Liu et al. [52] demonstrated that prolonged low-temperature exposure increased reliance on protein metabolism, as evidenced by marked shifts in plasma urea and glycerol levels. Similarly, a liver proteomic study in gilthead sea bream exposed to decreasing temperatures from 18 to 11 °C reported enhanced catabolic processes, including protein, together with alterations in amino acid metabolism during cold exposure [53]. In the present study, although temporal differences were observed between body sizes in the SC group, the crude protein content declined over the experimental period, whereas the lipid content increased in the subadult and juvenile fish. The decrease in whole body protein content may reflect reduced protein deposition associated with suppressed growth and temperature-induced feed restriction, together with increased utilization of endogenous protein under prolonged energy limitation. The concurrent increase in the lipid content may indicate that protein was relatively more extensively utilized than lipid during overwintering in the SC group. Such changes in body composition are consistent with alterations in protein and lipid metabolism reported under prolonged cold exposure [27,52,53] and may reflect both adaptive adjustments and metabolic constraints under chronic low temperature conditions.

Chronic exposure to low water temperatures can induce reactive oxygen species generation, which results in oxidative stress in fish. SOD constitutes the first line of antioxidant defense by converting superoxide radicals (O_2_^−^) into H_2_O_2_, which is subsequently detoxified by CAT and GPX [35,54]. While CAT rapidly decomposes H_2_O_2_ at high concentrations, GPX plays a crucial role in scavenging both H_2_O_2_ and lipid peroxides, thereby protecting cellular membranes from oxidative damage [55]. Therefore, increases in these antioxidant parameters could serve as important indicators for assessing the physiological stress responses of fish and their capacity to regulate and mitigate environmental stress. However, hepatic antioxidant enzymes (GPX, CAT, and SOD) generally remained stable across groups throughout the period, suggesting that threeline grunt maintained metabolic homeostasis without experiencing substantial oxidative stress under the given conditions. Supporting this interpretation, the lack of clear trends in plasma cortisol and hepatic HSP-70 levels suggested that the winter temperature environment during the experimental period did not impose sufficient stress responses or cellular damage to the liver. Although MDA, a byproduct of lipid peroxidation and an indicator of oxidative damage, was significantly higher in subadult fish in the SC group than in the IT group in April, the data obtained in the present study could not reveal the specific cause for this increase. The elevated MDA levels may have been influenced by factors other than water temperature, including seasonal changes in feeding conditions, physiological status, or other environmental factors. In addition, the seasonal increase in water temperature may have contributed to increased metabolic activity and oxidative burden [56]. However, the relative contributions of these factors remain unclear and warrant further investigation.

PCA was conducted to reduce dimensionality while minimizing information loss, and to explore relationships among the experimental groups and correlations among the biochemical, antioxidant, and stress-related parameters. PCA was performed separately for subadult and juvenile fish. Although certain physiological parameters might be expected to show coherent covariation under winter temperature conditions, several parameters were distributed across PC1 and PC2 rather than showing consistently high loadings on a single PC. In addition, the relatively low explanatory power and lack of distinct clustering among the experimental groups suggested that the physiological responses during overwintering were not characterized by a single consistent pattern, but may have varied across multiple physiological pathways, unlike in previous studies [57,58]. Similarly, hierarchical clustering showed no clear environment-specific clustering in subadult fish, whereas the juvenile fish tended to form separate clusters according to rearing environment from February to April. This clustering tendency suggests that body size could have contributed to differences in responses to rearing conditions, potentially reflecting differences in physiological regulation and adaptation to changing environmental conditions.

Overall, the size-dependent physiological disparities observed in our study highlight distinct overwintering strategies and energetic constraints in this species. The substantially lower survival, marked growth depression, and CF reduction in juveniles indicate that limited energy storage and constrained stress coping capacity appear to impose a significant burden on smaller fish [59]. The rapid decline in tissue proximate composition suggested that smaller energy reserves in juveniles lead to earlier metabolic exhaustion during winter season [60]. Furthermore, their compromised physiological status implied that less developed antioxidant defenses and heightened stress responses render juveniles particularly vulnerable to oxidative damage and metabolic stress [59]. Consequently, whereas subadults effectively mobilized larger reserves to maintain physiological homeostasis, juveniles reached critical energetic limits that endanger overwinter survival [61]. To overcome these size-dependent constraints, practical management strategies, such as supplying lipid-enriched diets to build pre-winter energy reserves or advancing early-season seed production, can be considered, by further validation through future studies remains necessary.

## 5. Conclusions

This study provides a field-scale assessment of the overwintering responses of subadult and juvenile threeline grunt reared in sea cages along the southern coast of Korea. Fish maintained in sea cages showed lower survival and growth performance than those maintained in inland tanks, together with changes in biological indices, body composition, and several biochemical parameters during the winter period. Nevertheless, more than 75% of both size groups survived the overwintering period in sea cages, whereas the measured antioxidant and stress-related parameters did not exhibit a consistently coordinated response. Juveniles showed greater reduction in several performance indicators than subadults, suggesting that body size should be considered when developing overwintering management strategies. These findings provide preliminary information on the biological capacity of threeline grunt to overwinter under sea-cage conditions in southern Korea. However, because the study lacked independent rearing-unit replication and used a relatively limited number of analytical replicates, further replicated studies are required before the commercial feasibility of sea-cage overwintering can be determined. Future research should evaluate practical overwintering strategies, including pre-winter nutritional management with lipid-enriched diets, earlier seed production to achieve larger body size before winter, appropriate cage-depth adjustment, and indoor pre-overwinter acclimation, to improve overwintering performance. In addition, year-round rearing performance and economic feasibility should be evaluated to further assess the potential of this species for commercial aquaculture.

## Figures and Tables

**Table 1:** Survival and growth performance of differently sized threeline grunt (*Parapristipoma trilineatum*) reared in inland and sea cages during the experimental period (from January to April).

Experimental Group		Survival Rate (%)	Weight Gain (%)	SGR_w_^1^ (%/Day)	SGR_L_^2^ (%/Day)	TGC ^3^
Fish Size	Rearing Site					
Subadult	Inland tank	92.0	15.4	0.113	0.077	0.133
	Sea cage	77.2	2.8	0.013	0.061	0.026
Juvenile	Inland tank	96.3	21.8	0.200	0.054	0.148
	Sea cage	75.8	0.9	0.002	0.021	0.004

^1^ Specific growth rate of weight (SGR_W_, %/day) = (Ln final weight of fish − Ln initial weight of fish) × 100/days of feeding trial. ^2^ Specific growth rate of length (SGR_L_, %/day) = (Ln final standard length of fish − Ln initial standard length of fish) × 100/days of feeding trial. ^3^ Thermal growth coefficient (TGC) = (final weight of fish^1/3^ − initial weight of fish^1/3^)/days of feeding trial/average temperature × 1000.

**Table 2:** Monthly changes in proximate composition (% wet weight) of the whole body of the threeline grunt (*Parapristipoma trilineatum*) during the experimental period.

Month	Subadult-Moisture			Juvenile-Moisture		
	Inland Tank	Sea Cage	I vs. S	Inland Tank	Sea Cage	I vs. S
Jan.	65.0 ± 0.51	64.5 ± 0.23 ^a^	ns	63.9 ± 0.20	65.1 ± 0.35	*
Feb.	66.3 ± 0.26	62.0 ± 0.74 ^b^	*	63.7 ± 1.52	64.4 ± 0.36	ns
Mar.	65.6 ± 0.61	63.4 ± 0.52 ^ab^	*	64.8 ± 0.24	63.9 ± 0.47	ns
Apr.	65.8 ± 0.60	64.5 ± 0.63 ^a^	ns	64.4 ± 0.28	64.4 ± 0.54	ns
*p*-value	*p* = 0.377	*p* = 0.025		*p* = 0.738	*p* = 0.342	
Month	Subadult-crude protein			Juvenile-crude protein		
	Inland tank	Sea cage	I vs. S	Inland tank	Sea cage	I vs. S
Jan.	19.8 ± 0.14	19.2 ± 0.17 ^a^	*	18.9 ± 0.13 ^A^	18.1 ± 0.27 ^a^	ns
Feb.	19.8 ± 0.13	18.0 ± 0.17 ^b^	*	18.0 ± 0.31 ^AB^	17.9 ± 0.34 ^ab^	ns
Mar.	19.7 ± 0.37	17.8 ± 0.42 ^b^	*	18.0 ± 0.39 ^AB^	17.1 ± 0.16 ^b^	ns
Apr.	18.5 ± 0.46	18.5 ± 0.15 ^ab^	ns	17.6 ± 0.10 ^B^	16.8 ± 0.15 ^b^	*
*p*-value	*p* = 0.1	*p* = 0.009		*p* = 0.026	*p* = 0.007	
Month	Subadult-crude lipid			Juvenile-crude lipid		
	Inland tank	Sea cage	I vs. S	Inland tank	Sea cage	I vs. S
Jan.	9.5 ± 0.74	11.1 ± 0.48 ^b^	ns	11.7 ± 0.11	11.5 ± 0.20 ^b^	ns
Feb.	8.3 ± 0.39	15.1 ± 1.00 ^a^	*	13.5 ± 1.33	12.3 ± 0.35 ^ab^	ns
Mar.	9.8 ± 1.10	13.7 ± 0.87 ^ab^	*	11.7 ± 0.46	13.3 ± 0.70 ^ab^	ns
Apr.	9.9 ± 0.62	11.3 ± 1.07 ^b^	ns	12.8 ± 0.13	13.8 ± 0.59 ^a^	ns
*p*-value	*p* = 0.423	*p* = 0.02		*p* = 0.253	*p* = 0.03	
Month	Subadult-ash			Juvenile-ash		
	Inland tank	Sea cage	I vs. S	Inland tank	Sea cage	I vs. S
Jan.	5.9 ± 0.34	5.5 ± 0.20	ns	5.6 ± 0.17	5.2 ± 0.16	ns
Feb.	5.8 ± 0.34	4.8 ± 0.19	ns	4.8 ± 0.13	5.1 ± 0.20	ns
Mar.	5.3 ± 0.27	5.0 ± 0.12	ns	5.2 ± 0.24	5.2 ± 0.14	ns
Apr.	5.5 ± 0.27	5.7 ± 0.30	ns	5.1 ± 0.20	4.9 ± 0.05	ns
*p*-value	*p* = 0.404	*p* = 0.11		*p* = 0.082	*p* = 0.359	

Values (means of four replicate ± SE) in the same column sharing a common superscript letter (uppercase for the Inland tank group and lowercase for the Sea cage group, respectively) are not significantly different (*p* > 0.05) in each group. The asterisks (*) indicate significant differences (*p* < 0.05) and “ns” indicates no significant (*p* > 0.05) differences between the Inland tank group and the Sea cage group in each sampling time.

## Data Availability

On reasonable request, the authors will provide all necessary data.
